# The environmental and health impacts of tobacco agriculture, cigarette manufacture and consumption

**DOI:** 10.2471/BLT.15.152744

**Published:** 2015-10-22

**Authors:** Thomas E Novotny, Stella Aguinaga Bialous, Lindsay Burt, Clifton Curtis, Vera Luiza da Costa, Silvae Usman Iqtidar, Yuchen Liu, Sameer Pujari, Edouard Tursan d'Espaignet

**Affiliations:** a Graduate School of Public Health, San Diego State University, San Diego, United States of America (USA).; b School of Nursing, University of California San Francisco, San Francisco, USA.; c Maxwell School Citizenship and Public Affairs, Syracuse, New York, USA.; d The Cigarette Butt Pollution Project, Washington, USA.; e Secretariat for the WHO Framework Convention on Tobacco Control, Geneva, Switzerland.; f Simon Fraser University, Vancouver, Canada.; g Tobacco Free Initiative, World Health Organization, avenue Appia 20, 1211 Geneva 27, Switzerland.

## Abstract

The health consequences of tobacco use are well known, but less recognized are the significant environmental impacts of tobacco production and use. The environmental impacts of tobacco include tobacco growing and curing; product manufacturing and distribution; product consumption; and post-consumption waste. The World Health Organization’s Framework Convention on Tobacco Control addresses environmental concerns in Articles 17 and 18, which primarily apply to tobacco agriculture. Article 5.3 calls for protection from policy interference by the tobacco industry regarding the environmental harms of tobacco production and use. We detail the environmental impacts of the tobacco life-cycle and suggest policy responses.

## Résumé

Les conséquences du tabagisme sur la santé sont notoires. En revanche, les impacts environnementaux considérables de la production et de la consommation de tabac sont moins connus. Ces impacts environnementaux sont liés à la culture et au séchage du tabac, à la fabrication des produits du tabac et à leur distribution, au tabagisme et aux déchets générés après consommation. La Convention-cadre de l’OMS pour la lutte antitabac évoque ces problèmes environnementaux dans ses Articles 17 et 18, qui s'appliquent avant tout à la culture du tabac. L'Article 5.3 préconise de ne pas laisser l'industrie du tabac influencer les mesures politiques en ce qui concerne les effets négatifs de la production et de la consommation du tabac sur l'environnement. Nous détaillons dans ce dossier les impacts environnementaux sur tout le cycle de vie du tabac et formulons plusieurs suggestions en termes de réponse politique.

## Resumen

Las consecuencias sanitarias del consumo de tabaco son bien conocidas, pero no tanto los significativos efectos que el cultivo y consumo de tabaco tienen en el medio ambiente. Los efectos medioambientales del tabaco incluyen el crecimiento y la cura del tabaco, la producción y distribución del producto, el consumo del producto y los residuos resultantes de su consumo. El Convenio Marco de la OMS para el Control del Tabaco aborda las preocupaciones medioambientales en los Artículos 17 y 18, los cuales se aplican principalmente en el cultivo del tabaco. El Artículo 5.3 exige medidas cautelares respecto a las políticas de interferencia de la industria del tabaco en lo que se refiere a los daños medioambientales del cultivo y el consumo de tabaco. Se enumeran los efectos medioambientales del ciclo de vida del tabaco y se sugieren respuestas políticas.

## ملخص

إن الآثار الصحية لاستخدام التبغ معروفة جيدًا، ولكن المعرفة بالتأثيرات البيئية الكبرى لإنتاج التبغ واستخدامه ليست بذات القدر من الانتشار. وتشتمل الآثار البيئية للتبغ على زراعة التبغ ومعالجته؛ وصناعة المنتجات وتوزيعها واستهلاكها؛ والنفايات الناتجة بعد الاستهلاك. وتتناول الاتفاقية الإطارية لمنظمة الصحة العالمية المعنية بمكافحة التبغ المخاوف البيئية في المادتين 17 و18، واللتين تنطبقان بشكل أساسي على زراعة التبغ. وتدعو المادة 5-3 للحماية من التدخل في السياسات من جانب قطاع صناعة التبغ فيما يتعلق بالأضرار البيئية لإنتاج التبغ واستخدامه. ونحن نتناول بالتفصيل التأثيرات البيئية لدورة حياة التبغ كما نقترح الاستجابات السياسية.

## 摘要

使用烟草对健康造成的后果已经众所周知，但是生产和使用烟草对环境产生的重大影响却鲜为人知。烟草对环境的影响包括：烟草种植和烘焙、产品制造和分销、产品消费和消费后产生的垃圾。世界卫生组织发布的《烟草控制框架公约》第 17 条和第 18 条中强调的环境问题主要适用于烟草农业。关于烟草生产和使用对环境造成的伤害，第 5.3 款中呼吁烟草行业采取政策干预来保护环境。我们将会详细探讨烟草在其生命周期内对环境造成的影响，同时建议政策应对。

## Резюме

Влияние табака на здоровье изучено хорошо, но куда менее известно значительное воздействие производства и потребления табака на окружающую среду. Воздействие табака на окружающую среду происходит во время выращивания и сушки табака, изготовления, распределения и потребления табачных изделий; также влияние на окружающую среду оказывают отходы потребления. В Рамочной конвенции Всемирной организации здравоохранения по борьбе против табака проблемам окружающей среды посвящены статьи 17 и 18, в которых внимание уделяется в основном сельскохозяйственному производству табака. В статье 5.3 отмечается необходимость защиты от вмешательства табачной промышленности в политику здравоохранения в плане причинения вреда окружающей среде в результате производства и потребления табачных изделий. Далее подробно описывается влияние жизненного цикла табачных изделий на окружающую среду и предлагаются ответные меры в области политики.

## Introduction

The human health impacts of tobacco use are well-documented. The World Health Organization (WHO) estimates that there will be more than 8 million tobacco-related deaths a year by 2030, amounting to 10% of annual deaths worldwide.^1^

The impact that tobacco has on the environment is less well recognized. The WHO Framework Convention on Tobacco Control (FCTC) addresses the environmental concerns regarding tobacco in Article 18, which states that:

“In carrying out their obligations under this Convention, the Parties agree to have due regard to the protection of the environment and the health of persons in relation to the environment in respect of tobacco cultivation and manufacture within their respective territories.”

In response, a series of policy options and recommendations were agreed at the sixth Conference of the Parties to the FCTC in 2014.^2^ The meeting identified key sources of environmental concern and recommended environmental impact studies on tobacco growing.^2^ Given the environmental and occupational health concerns associated with tobacco growing, the FCTC also addresses the need for alternative livelihoods for tobacco growers in Article 17.

The environmental lifecycle of tobacco can be roughly divided into four stages: (i) tobacco growing and curing; (ii) product manufacturing and distribution; (iii) product consumption; and (iv) post-consumption waste. Here, we describe the environmental and health concerns at each of these stages and propose recommendations for policy-makers.

### Tobacco growing and curing

In 2011, around 4 200 000 hectares of land were devoted to tobacco growing, representing less than 1% of total arable land globally; however, in several low- and middle-income countries, the percentage of arable land devoted to tobacco growing has recently increased.^1^ For example, it has almost doubled in China, Malawi and the United Republic of Tanzania since the 1960s. Deforestation for tobacco growing has many serious environmental consequences – including loss of biodiversity, soil erosion and degradation, water pollution and increases in atmospheric carbon dioxide.

Tobacco growing usually involves substantial use of chemicals – including pesticides, fertilizers and growth regulators.^3^ These chemicals may affect drinking water sources as a result of run-off from tobacco growing areas. Research has also shown that tobacco crops deplete soil nutrients by taking up more nitrogen, phosphorus and potassium than other major crops. This depletion is compounded by topping and de-suckering plants, which increase the nicotine content and leaf yields of tobacco plants.^3^

Land used for subsistence farming in low- and middle-income countries may be diverted to tobacco as a cash crop. Intensive lobbying and investments by multinational tobacco companies (e.g. Philip Morris International, British American Tobacco and Japan Tobacco International) and leaf buyers (e.g. Universal Corporation and Alliance One International) along with market liberalization measures have encouraged the expansion of tobacco agriculture in low- and middle-income countries. Many of these countries have limited legislative and economic capacities to resist multinational tobacco companies’ influence and investments. As a consequence of expanded tobacco agriculture, there are short-term economic benefits for some farmers, but there will be long-term social, economic, health and environmental detriments for many others.^4^

Due to widespread concerns about unfair labour practices in tobacco agriculture, tobacco control advocates have recently been working with tobacco farmers and farm workers to ensure the right to collective bargaining and to receive living wages and fair leaf prices.^5^ Given the agricultural labour practices in both low- and middle-income countries and more developed countries, attention is also needed to ensure the safety of children involved in tobacco farming. Farm workers, especially child labourers, minorities and migrant workers are at risk of nicotine toxicity (green tobacco illness), caused by handling tobacco leaves without protection during harvest and processing.^6^

### Manufacturing and distribution

In 1995, it was estimated that global tobacco manufacturing produced over 2 000 000 tonnes of solid waste, 300 000 tonnes of non-recyclable nicotine-containing waste and 200 000 tonnes of chemical waste.^7^ If annual cigarette production had remained constant for the past 20 years (output has actually increased from 5 to 6.3 trillion cigarettes annually), tobacco factories would have deposited a total of 45 000 000 tonnes of solid wastes, 6 000 000 tonnes of nicotine waste and almost 4 000 000 tonnes of chemical wastes during this time. Other toxic by-products of tobacco manufacturing or chemicals used in manufacturing include ammonia, hydrochloric acid, toluene and methyl ethyl ketone.

### Product consumption

The health impacts of environmental tobacco smoke exposure include lung cancer, cardiovascular disease and pulmonary disease.^8^ Exposure to residual chemicals in environments where smoking has taken place may also have human health impacts, though these impacts have not yet been quantified.^9^Most cigarettes are lit using matches or gas-filled lighters. If, for example, one wooden match is used to light two cigarettes, the six trillion cigarettes smoked globally each year would require the destruction of about nine million trees to produce three trillion matches.^10^ There are also environmental impacts of manufacturing and disposing of the plastic, metal and butane used in making cigarette lighters.

Cigarettes remain an important cause of accidental fires and resulting deaths. In the United Kingdom of Great Britain and Northern Ireland, cigarettes caused 7% of fires in 2013–2014, making them the single most important cause of deaths related to fires (34 deaths/1000 fires).^11^ In the United States of America, cigarettes have been responsible for 8–10% of all fires over the past 10 years (on average 90 000 fires per year); they also remain the single most important cause of deaths related to fires (540 of 2855 total deaths in 2011).^12^ These fires were responsible for 621 million United States dollars in direct property damage and 1640 civilian injuries. Regulations requiring cigarettes to self-extinguish in Canada and the USA were associated with a 30% decline in fire-related deaths from 2003 to 2011.^13^

### Post-consumption waste

Cigarette butts are the most commonly discarded piece of waste globally and are the most frequent item of litter picked up on beaches and water edges worldwide.^14^ The non-biodegradable cellulose acetate filter attached to most manufactured cigarettes is the main component of cigarette butt waste and trillions of filter-tipped butts are discarded annually. Assuming that each filter weighs 170 milligrams, the weight of all tobacco-attributable non-biodegradable (filter) waste discarded annually is about 175 200 tonnes.

Hazardous substances have been identified in cigarette butts – including arsenic, lead, nicotine and ethyl phenol. These substances are leached from discarded butts into aquatic environments and soil. Although the environmental impact of this waste has not yet been quantified, the large quantity of discarded butts may allow leachates to affect the quality of drinking water. Other post-consumption wastes, such as medicines, pesticides and plastic microbeads from cosmetics, have been found in drinking water sources.^15^^–^^17^ It is possible that tobacco product waste may also prove to be a significant environmental contaminant and potential human health hazard through bioaccumulation in the food-chain.

With 6 trillion cigarettes manufactured annually, about 300 billion packages (assuming 20 cigarettes per pack) are made for tobacco products. Assuming each empty pack weighs about six grams, this amounts to about 1 800 000 tonnes of packaging waste, composed of paper, ink, cellophane, foil and glue. The waste from cartons and boxes used for distribution and packing brings the total annual solid post-consumption waste to at least 2 000 000 tonnes. This compares with an estimated 1 830 000 tonnes annually of plastic waste from mineral water bottles (estimation method available from the corresponding author).

Electronic cigarettes may contain batteries that require special disposal as well as chemicals, packaging and other non-biodegradable materials. The US Federal Emergency Management Agency (FEMA) has expressed concerns about the flammability and lack of product regulation of electronic cigarettes and their components.^18^

### Carbon dioxide emissions

Tobacco smoking leads directly to the emission of 2 600 000 tonnes of carbon dioxide and about 5 200 000 tonnes of methane.^19^Data from 66 low- and middle-income countries showed that tobacco growing and curing caused significant deforestation between 1990 and 1995, amounting to approximately 2000 hectares – on average, 5% of each country’s estimated deforestation during that five-year period.^20^ Worldwide, approximately 13 000 000 hectares of forest are lost due to agriculture or natural causes each year,^21^ and of this, at least 200 000 hectares are for tobacco agriculture and curing.^1^ Deforestation is the second largest anthropogenic source of carbon dioxide to the atmosphere (approximately 20%), after fossil fuel combustion.^22^ One estimate of the impact of deforestation in tobacco agriculture and curing is that it causes almost 5% of global greenhouse gas production.^23^

Despite their now well-known efforts to sow doubt among the public and policy-makers about anthropogenic climate change,^24^ tobacco companies have advertised their efforts to reduce carbon emissions. British American Tobacco estimated in 2006 that production of one million cigarettes produces 0.79 tonnes of carbon dioxide. According to this estimate, 4 740 000 tonnes of carbon dioxide would be emitted annually by global cigarette manufacturing. Other analyses assert that this is a gross underestimate of the greenhouse gas burden due to tobacco growing, manufacturing and transport.^23^ No estimates are as yet available on the extent of carbon dioxide emissions due to tobacco product transport.

## Proposed next steps

The FCTC recommendations encompass all aspects of the livelihoods of tobacco growers and workers – including health, economic, social, environmental and food security concerns.^25^ The recommendations re-emphasized the need to confront the vested interests of tobacco companies. These companies have promoted policies that avoid all environmental responsibility of the producer, and they attempt to divert public attention away from their environmental responsibilities through corporate social responsibility programmes.^26^ Protecting the public against the tobacco industry’s environmental impact is aligned with FCTC Article 5.3 and its guidelines, which remind Parties that:

“There is a fundamental and irreconcilable conflict between the tobacco industry’s interests and public health policy interests.”^27^

The FCTC recommendations also propose conducting an analysis of the main barriers and existing opportunities for Article 18 implementation.^2^

A community of concern needs to be established among multiple sectors – including health, agriculture, trade and environment – to address the environmental impacts of tobacco production and use. The FCTC Parties may consider such a broad approach as a new way to include academia, nongovernmental organizations and non-party countries. It is clear that tobacco control intersects with other pressing global issues such as sustainable development, environmental policy, climate change, trade agreements and human rights. By taking broad-based but effective action against the environmental hazards created by the tobacco industry, the demand for tobacco products will be further reduced. With strengthened environmental policies, there will be increased costs for tobacco products, decreased social acceptance of tobacco use and changes in the most commonly used tobacco products.

Policy options and recommendations on alternatives to tobacco growing involve comprehensive, environmentally-oriented tobacco control interventions for both tobacco growing and non-growing countries. We propose seven recommendations for Parties to the FCTC to consider. First, identify, prevent, treat and monitor health effects related to tobacco growing among farmers and workers. Second, develop strategies to free tobacco farmers and especially their children from unfair and unsafe agricultural and labour-related practices. Third, strengthen regulation of tobacco agriculture to prevent deforestation and land degradation. Fourth, implement extended producer responsibility regulations on the tobacco industry to reduce, mitigate and prevent manufacturing and post-consumption tobacco product waste. Fifth, extend tobacco product sales regulation to eliminate single-use filters – including any biodegradable varieties – to reduce post-consumption waste. Sixth, engage litigation and economic interventions to recover the costs of industry misconduct and environmental damages. Seventh, innovate, improve and enforce new and existing environmental regulations and agreements that may apply to tobacco manufacturing, transport and management of post consumption waste.
